# Luteal phase ovarian stimulation for poor ovarian responders

**DOI:** 10.5935/1518-0557.20180045

**Published:** 2018

**Authors:** Wei Zhang, Meimei Wang, Shuang Wang, Hongchu Bao, Qinglan Qu, Ning Zhang, Cuifang Hao

**Affiliations:** 1 Yantai Yuhuangding Hospital of Qingdao University - Yantai - China

**Keywords:** Poor ovarian response, ovarian stimulation, live birth rate, IVF-ET

## Abstract

**Objective:**

To compare the clinical outcomes of follicular versus luteal phase ovarian stimulation in women with poor ovarian response (Bologna criteria) undergoing IVF.

**Methods:**

This retrospective study investigated 446 patients submitted to 507 cycles in three groups. First, the two larger cohorts were examined: 154 patients treated with luteal phase ovarian stimulation (Group Lu); and 231 patients administered follicular phase ovarian stimulation (Group Fo). Then the clinical outcomes of 61 patients submitted to double ovarian stimulation were analyzed. Clinical outcomes included number of retrieved oocytes, fertilization rate, cleavage rate, top-quality embryo rate, clinical pregnancy rate (CPR), and live birth rate (LBR).

**Results:**

Longer stimulation, higher dosages of HMG, and higher MII oocyte rates were achieved in Group Lu (*p*<0.001). There were no significant differences in CPR and LBR between the two groups offered frozen-thawed embryo transfer (28.4% vs. 33.0%, *p*=0.484; 22.9% vs. 25.5%, *p*=0.666). In the double ovarian stimulation group, the number of oocytes retrieved in the luteal phase stimulation protocol was higher (*p*=0.035), although luteal phase stimulation yielded a lower rate of MII oocytes (*p*=0.031). CPR and LBR were not statistically different (13.8% vs. 21.4%, *p*=0.525; 10.3% vs. 14.3%, *p*=0.706).

**Conclusion:**

Luteal phase ovarian stimulation may be a promising protocol to treat women with POR, particularly for patients unable to yield enough viable embryos through follicular phase ovarian stimulation or other protocols.

## INTRODUCTION

Ovarian stimulation improves the outcome of assisted reproductive technology (ART) treatments by increasing the number of oocytes and viable embryos. Unfortunately, the incidence of poor ovarian response (POR) ranges from 9% to 24% in women undergoing ovarian stimulation for ART (Ubaldi *et al.*, 2005). There is no perfect predictive test available to assess ovarian response or screening test for POR. Women with POR have the poorest prognosis for ovarian stimulation. One of the limitations in interpreting the relevant literature is the huge discrepancy in the definitions of POR (Polyzos & Devroey, 2011). Therefore, the ESHRE Working Group has proposed a definition for POR. The cause of POR may be associated with reduced ovarian reserve (Ferraretti *et al*., 2011). Though various ovarian stimulation protocols have been applied to improve ovarian response, POR is still a challenging condition for patients and clinicians (Venetis *et al*., 2010; Kryou *et al*., 2007).

Traditional minimal stimulation starts in the early follicular phase and relies on the physiological development of the endometrium to optimize the chances of embryo implantation (ET). Attempts to start stimulation at any time in the menstrual cycle (‘random-start’ protocols) rather than in the early follicular phase or after down regulation have been reported (Ozkaya *et al*., 2012). Double ovarian stimulation resulted in the retrieval of more oocytes within a short period of time and offered new hope for women with POR (Kuang *et al*., 2014a; Cardoso *et al.*, 2017). In order to explore the efficacy of luteal phase ovarian stimulation in women with POR, this study compared the clinical outcomes of luteal phase ovarian stimulation and follicular phase ovarian stimulation protocols.

## MATERIALS AND METHODS

### Study population

Patients seen between January 2013 and December 2014 were selected based on the Bologna criteria for POR. The included patients gave informed consent allowing the use of their clinical records. Patients with at least two of the following findings were diagnosed with POR: (i) advanced maternal age (≥40 years) or any other risk factor for POR; (ii) previous POR (≤3 oocytes from a conventional stimulation protocol); (iii) abnormal ovarian reserve test (i.e., antral follicle count (AFC) <5-7 follicles or anti-Müllerian hormone (AMH) <0.5-1.1 ng/ml) (Ferraretti *et al.*, 2011). The Institutional Ethical Review Board approved the study.

Patients with POR were preferentially offered follicular phase ovarian stimulation. Luteal phase ovarian stimulation was performed to increase the number of embryos or to enable the retrieval of oocytes in patients without oocytes after follicular phase ovarian stimulation. The study enrolled 446 patients with POR undergoing minimal stimulation during the follicular phase (292 cycles) and/or the luteal phase (215 cycles). All patients were offered their first cycle of treatment. Follicular stimulation alone was administered to 231 patients; 154 patients were given luteal phase stimulation only in 154 cycles; 61 patients received double stimulation in the same menstrual cycle (122 cycles). Follicular phase stimulation was carried out first and luteal phase stimulation was performed after oocyte retrieval.

### Luteal phase ovarian stimulation

A typical protocol for luteal phase ovarian stimulation starts 2-7 days following ovulation or oocyte retrieval. In our study, transvaginal ultrasound (Aloka ultrasound imaging system, Japan) examination was carried out two days after oocyte retrieval or ovulation. After verifying the presence of at least two antral follicles measuring 2-8 mm in diameter, the patients were given clomiphene citrate (CC) (Fertilan; Codal-Synto Ltd., France) 50-100 mg daily and human menopausal gonadotropin (HMG) (Livzon Pharmaceutical Group Co., Ltd., China) 75-150 IU daily. Minimal stimulation after ovulation was performed in 154 patients in Group Lu (154 cycles). Sixty-one patients started ovarian stimulation in the early follicular phase; luteal phase ovarian stimulation was initiated in these patients during the same menstrual cycle after oocyte retrieval (122 cycles). Duphaston (Abbott Biologicals B.V., America) 20 mg daily was added from the day of ovulation or first oocyte retrieval for luteal support and to postpone menstruation, avoid oocyte retrieval during menstruation, and prevent the risk of infection from the procedure.

### Follicular phase ovarian stimulation

Two hundred and thirty-one patients were screened by transvaginal ultrasound on day 3 of their menstrual cycle in Group Fo (231 cycles). Meanwhile, CC 50-100 mg daily was given from cycle days 3 to 7. HMG 75-150 IU daily was added starting on cycle day 8 and was administered until before trigger day. Meanwhile, serum concentrations of E_2_, LH, and P were measured.

When one or two dominant follicles measuring >18mm in diameter were observed, ovulation was triggered with 250µg of recombinant human chorionic gonadotropin (r-HCG) (Ovidrel^®^; Merck Serno, Germany). Oocyte retrieval was performed approximately 36 hours after hCG administration. Depending on the quality of the retrieved sperm, either conventional IVF or intra-cytoplasmic sperm injection (ICSI) was performed to fertilize the eggs. In cycles with luteal phase ovarian stimulation, competent embryos were cryopreserved for later transfer. In other cycles, the competent embryos were transferred on Day 3 or cryopreserved on account of endometrium thickness or by patient choice.

**Figure 1:**
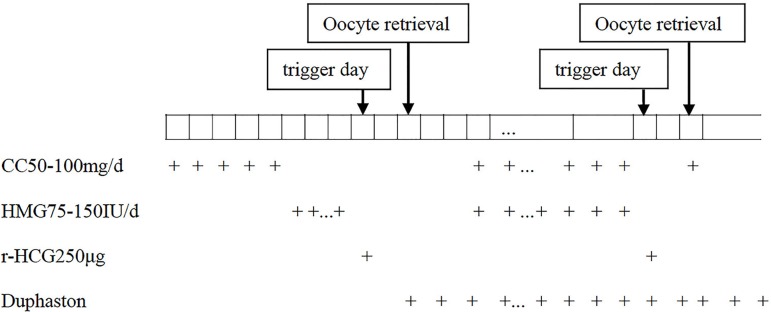
Double ovarian stimulation protocol during the follicular and luteal phases in patients with POR. CC, clomiphene citrate; HMG, human menopausal gonadotropin; r-HCG, recombinant human chorionic gonadotropin

Figure 1 describes the double stimulation protocol.

### Endometrial preparation and frozen-thawed embryo transfer (FET)

In natural FET cycles, follicular growth and endometrial thickness were monitored by transvaginal ultrasound. Three days after ovulation, if endometrial thickness was greater than 8 mm, 3-day-old embryos were transferred.

Patients with irregular menstrual cycles were given CC and HMG according to the follicular phase ovarian stimulation protocol described above. Luteal phase support was achieved with 400mg transvaginal progesterone soft capsule daily (Utrogestan; Besins Manufacturing Belgium, France) beginning on the day of ovulation.

**Figure 2:**
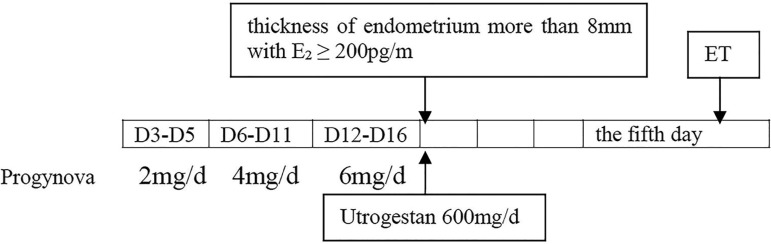
Hormone replacement therapy protocol in FET

Patients with irregular menstrual cycles or thin endometria during either natural or stimulated cycles were prescribed hormone replacement therapy. Oral estradiol valerate tablets (Progynova; Delpharm Lills S.A.S., Germany) 2mg daily from cycle days 3 to 5, 4mg daily from cycle days 6 to 11, and 6mg daily from cycle days 12 to 16 were administered. When the thickness of endometrium was greater than 8mm with E_2_ greater than 200pg/ml, 600mg transvaginal progesterone soft capsule daily was added (Figure 2). ET was carried out on the fifth day of luteal phase support. A maximum of two embryos were transferred per patient; the entire procedure was performed according to the manufacturer’s instructions.

Embryos were cryopreserved by vitrification (Rapidvit™ Cleave, Vitrolife Sweden AB Göteborg, Sweden). Warming was performed according to the manufacturer’s instructions using a RapidWarm™ Cleave kit (Vitrolife Sweden AB Göteborg, Sweden).

### Pregnancy confirmation

Clinical pregnancy was confirmed with ultrasound examination and the observation of a gestational sac with or without cardiac activity on week 7 of gestation. The rate of miscarriages per clinical pregnancy was defined as the proportion of clinically pregnant patients who failed to continue development to 28 weeks of gestation. Information on pregnancy outcomes was collected from all pregnant women by phone. Live birth was defined as the delivery of a fetus with signs of life.

### Statistical analysis

For each patient group, categorical data were presented in the form of number of cases and proportions. Continuous data were presented as median values and interquartile ranges. Student’s t-test and the chi-square test were used for data analysis. Statistical analysis was performed on SPSS 17.0 (SPSS Inc., Chicago, IL, USA). Differences with *p* <0.05 were deemed statistically significant.

## RESULTS

### Comparisons between groups Lu and Fo

There were no differences in AFC, BMI or baseline hormone levels (FSH, LH and E_2_) of the patients in the two groups (*p*＞0.05). However, the subjects in Group Lu were older than the patients in Group Fo (Table 1).

**Table 1:** Baseline characteristics of patients in Groups Lu and Fo.

	Group Lu	Group Fo	*p*-value
No. of cycles	154	231	-
Age (years)	39.1±4.8	37.4±4.8	0.002
BMI (Kg/m^2^)	23.5±3.4	24.0±3.0	0.206
Antral follicle count (AFC)	5.3±2.9	5.9±3.6	0.129
Baseline FSH (mIU/mL)	10.0±5.6	9.1±4.0	0.102
Baseline LH (mIU/mL)	5.2±2.2	5.1±2.9	0.701
Baseline E_2_ (pg/ml)	47.9±42.5	44.8±35.6	0.447

The values are presented as mean±standard deviation(SD).

NS = not significant;

No. = number.

Length of stimulation, dosage of HMG and MII oocyte rate in Group Lu were significantly higher than in Group Fo (*p*<0.001). There were no significant differences between the two groups on number of retrieved oocytes, fertilization rate, cleavage rate, and top-quality embryo rate (*P>*0.05).

To this point in time, 109 FET procedures have been carried out in Group Lu. Thirty-one patients became clinically pregnant yielding a CPR of 28.4%. Five miscarriages occurred, yielding a miscarriage rate of 16.1%. Twenty-five patients delivered babies, yielding a live birth rate of 22.9%. One patient underwent laparoscopic surgery for ectopic pregnancy.

In Group Fo, 79 patients had ET after oocyte retrieval. Among these patients, 31 were clinically pregnant (CPR: 39.2%). Nine miscarriages occurred before 28 weeks of gestation (miscarriage rate: 29.0%). Twenty patients gave birth (LBR: 25.3%). One patient had an ectopic pregnancy and was treated with laparoscopic surgery. And one patient underwent a mid-trimester induction of labor because of a fetal anomaly. To this point in time, 94 FET procedures have been conducted. Thirty-one patients became clinically pregnant (CPR: 33.0%). Six miscarriages were recorded (miscarriage rate: 19.4%). Twenty-four delivered babies (LBR: 25.5%). One patient had an ectopic pregnancy and was given laparoscopic surgery.

There were no significant differences in CPR, miscarriage rate or LBR between the two groups offered FET (28.4% vs. 33.0%, *p*=0.484; 16.1% vs. 19.4%, *p*=0.740; 22.9% vs. 25.5%, *p*=0.666). Besides, there were no significant differences in CPR, miscarriage rate or LBR between ET and FET cycles in Group Fo (39.2 vs. 33.0%, *p*=0.392; 29.0% vs. 19.4%, *p*=0.374; 25.3% vs. 25.5%, *p*=0.974) (Table 2).

**Table 2:** Outcomes in Groups Lu Group Fo.

	Group Lu	Group Fo	*p*-*value*
Length of stimulation (days)	11.3±3.6	8.1±2.8	<0.001
Dosage of HMG(IU)	2336.8±848.8	1388.2±731.8	<0.001
No. of retrieved oocytes	2.7±2.1	2.4±1.5	0.085
Metaphase II oocyte rate *n* (%)	325/416 (78.1)	374/551 (67.9)	<0.001
Fertilization rate *n* (%)	349/416 (83.9)	441/551 (80.0)	0.125
Cleavage rate *n* (%)	345/349 (98.9)	437/441 (99.1)	0.739
Top-quality embryo rate *n* (%)	211/307 (68.7)	255/382 (66.8)	0.582
ET clinical pregnancy rate *n* (%)	-	31/79 (39.2)^a^	0.392
FET clinical pregnancy rate *n* (%)	31/109 (28.4)	31/94 (33.0)	0.484
ET miscarriage rate *n* (%)	-	9/31 (29.0)^b^	0.374
FET miscarriage rate *n* (%)	5/31 (16.1)	6/31 (19.4)	0.740
ET live birth rate *n* (%)	-	20/79 (25.3)^c^	0.974
FET live birth rate *n* (%)	25/109 (22.9)	24/94 (25.5)	0.666

NS = not significant; No.= number;

a = comparison of clinical pregnancy rates between ET cycles and FET cycles in Group Fo;

b = comparison of miscarriage rates between ET cycles and FET cycles in Group Fo;

c = comparison of live birth rates between ET cycles and FET cycles in Group Fo.

### Comparison of the two protocols in the same patients with POR

There were no significant differences in length of stimulation and dosage of HMG between the Lu and Fo protocols performed in the same patients (*p*=0.190, *p*=0.250). The number of retrieved oocytes in luteal phase ovarian stimulation was significantly higher than in follicular phase ovarian stimulation (*p*=0.035). The MII oocyte rate was lower in the luteal phase ovarian stimulation protocol (*p*=0.031). Cleavage and top-quality embryo rates were not statistically different (*p*=0.273, *p*=0.923) (Table 3).

**Table 3:** Comparison of the same patients undergoing Luteal phase ovarian stimulation protocol (Lu protocol) and Follicular phase ovarian stimulation protocol (Fo protocol).

	Lu protocol	Fo protocol	*p*-*value*
No. of cycles	61	61	-
Age (years)	39.9±4.7	39.9±4.6	1.000
Length of stimulation (day)	6.9±4.1	7.9±4.0	0.190
Dosage of HMG(IU)	1428.0±1043.3	1241.7±675.8	0.250
No. of retrieved oocytes	1.8±1.1	1.3±0.9	0.035
Metaphase II oocyte rate *n* (%)	72/99 (72.7)	57/65 (87.7)	0.031
Fertilization rate *n* (%)	86/99 (86.9)	51/65 (78.5)	0.156
Cleavage rate *n* (%)	84/86 (97.7)	51/51 (100)	0.273
Top-quality embryo rate *n* (%)	47/68 (69.1)	28/40 (70.0)	0.923
FET clinical pregnancy rate *n* (%)	4/29 (13.8)	3/14 (21.4)	0.525
FET miscarriage rate *n* (%)	1/4 (25)	0/3 (0)	0.350
FET live birth rate *n* (%)	3/29 (10.3)	2/14 (14.3)	0.706

NS = not significant;

No. = number.

Forty-three patients had completed FET by the end of the study period. Twenty-nine cycles involved embryos transferred from luteal phase ovarian stimulation. Four patients were clinically pregnant (CPR: 13.8%). One patient had a miscarriage (miscarriage rate: 25.0%). Three patients delivered babies (LBR: 10.3%). Fifteen FET cycles were conducted in patients with embryos from follicular phase ovarian stimulation. Three patients were clinically pregnant (CPR: 21.4%). Two patients delivered babies (LBR: 14.3%). There were no statistical differences in CPR or LBR (13.8% vs. 21.4%, *p*=0.525; 10.3% vs. 14.3%, *p*=0.706) (Table 3).

## DISCUSSION

The primary objective of stimulation protocols is to yield more oocytes, produce more viable embryos, and increase the probability of pregnancy. The quantities of oocytes and embryos appear to be recurrent variables influencing CPR (van Loendersloot *et al*., 2010; Cai *et al*., 2011; Choi *et al*., 2013). It has been reported that mature oocyte counts of 5-15 may lead to good clinical outcomes in IVF. Oocyte counts of fewer than five may represent significantly reduced CPR and LBR when compared to normal responders to ovarian stimulation (De Vries *et al*., 1999; Sharma *et al*., 2002). Patients with POR are known to have higher cycle cancellation and lower pregnancy rates. Several methods have been proposed to improve the pregnancy rates of these patients, but none has yielded promising results.

For the last two decades, the long protocol using GnRH agonists has been considered the standard method. Nevertheless, its potential may be limited for individuals with POR. Yoo *et al*. (2011) reported that mild stimulation resulted in similar clinical outcomes and yielded slightly better pregnancy rates than conventional ovarian stimulation in patients with POR aged 37 or older. For poor ovarian responders, the long protocol did not lead to better outcomes than luteal phase ovarian stimulation in terms of top-quality embryos. By its turn, mild stimulation for poor ovarian responders decreased the stimulating effect of gonadotropins in the ovaries and reduced patient discomfort and the cost of treatment. Luteal phase ovarian stimulation might be considered an option to poor ovarian responders without viable embryos.

Conventionally, minimal stimulation is initiated at the early follicular phase. The ‘random-start’ protocol overturned traditional ideas about minimal stimulation. Kuang *et al*. (2014b) reported that double stimulation during the follicular and luteal phases might provide a promising alternative or a rescue procedure for patients. In ovarian stimulation, the development of multiple follicles increases estrogen levels, usually resulting in a premature surge in LH levels caused by positive feedback. However, progesterone in high levels suppresses LH through negative feedback during luteal phase ovarian stimulation, in a process considered beneficial for the development of follicles. In this regard, the embryos originating from luteal phase ovarian stimulation might offer good development potential as illustrated in the CPR and LBR seen in FET. This finding is in agreement with previous studies. Therefore, luteal phase ovarian stimulation might provide more opportunities to retrieve oocytes within a short period of time in patients with POR, with the resulting embryos presenting similar development potential.

The aim of this study was to examine the clinical outcomes of patients with POR undergoing ART, particularly in terms of LBR - the most relevant indicator for patients and clinicians. Aging leads to the physiological decline of the ovarian follicle pool; the prevalence of POR is known to increase with age (Ferraretti *et al*., 2011). In this study, double stimulation revealed that luteal phase ovarian stimulation yielded more oocytes and similar top-quality embryo rates, CPR, and LBR in patients undergoing FET. Although older, the patients in Group Lu had more MⅡoocytes retrieved. Having greater numbers of oocytes retrieved within a shorter period of time might alleviate the psychological burden inherent to undergoing disappointing IVF procedures.

Buendgen *et al*. (2013) reported that the mean dose of HMG per oocyte retrieved from luteal phase stimulation was nearly twice the dosage prescribed in traditional protocols. Besides, ovarian sensitivity to HMG was significantly reduced during luteal phase ovarian stimulation. One of the impacting factors was pituitary suppression of co-existing high progesterone levels during the luteal phase (Kuang *et al*., 2014b). Another report showed that progesterone alone was not effective in inducing an endogenous gonadotropin surge or final oocyte maturation (Ozkaya *et al*., 2012). In our study, the length of stimulation and dosage of HMG in luteal phase stimulation were higher than in follicular phase ovarian stimulation. This observation further supports this idea.

It is usually necessary to accumulate viable embryos from several oocyte retrieval events (Cobo *et al*., 2012). Random-start controlled ovarian stimulation is as effective as conventional-start ovarian stimulation in fertility preservation (Moffat *et al*., 2014). Our study indicated that after ovulation or oocyte retrieval the antral follicles make up an exciting potential target to extend ovarian stimulation and perform one additional oocyte retrieval procedure. CC, a drug that increases pituitary FSH secretion by reducing negative estrogen feedback, has been widely used in controlled ovarian stimulation. CC reportedly causes endometrial thinning in 15-50% of patients because of its anti-estrogen effect, resulting in lower pregnancy rates (Mitwally & Casper, 2003). Therefore, patients with thin endometria (≤7mm) were advised to have their embryos frozen for later transfers. A number of ovarian stimulation, cryopreservation, and FET protocols with proven increased implantation and pregnancy rates and superior birth outcomes offer promising possibilities for poor ovarian responders (Cohen & Alikani, 2013). Moreover, embryo cryopreservation may provide ample time to get the endometrium ready for FET.

In the Bologna criteria, AFC and AMH were used as indicators of ovarian reserve. The two are deemed the most informative biomarkers of ovarian reserve (Broer *et al*., 2013). Kim *et al*. (2015) suggested that serum AMH alone is a sufficient predictor of POR. Unfortunately, serum AMH was not measured in this retrospective study. These factors may be evaluated in more detail in future targeted prospective studies. Although AMH might compromise pregnancy outcomes, lower levels of AMH do not impair embryo developmental competence (Borges *et al.*, 2017). As reported, women suspected for POR had lower live birth and cumulative live birth rates than normal ovarian responders; nonetheless, they were able to achieve reasonable outcomes and IVF treatment should not be precluded (Chai *et al*., 2015).

In conclusion, luteal phase ovarian stimulation might be a realistic option to produce more embryos within shorter periods of time for individuals with recurring failed oocyte retrieval procedures or patients without viable embryos administered conventional stimulation.
